# P1 and N1 Characteristics in Individuals with Normal Hearing and Hearing Loss, and Cochlear Implant Users: A Pilot Study

**DOI:** 10.3390/jcm13164941

**Published:** 2024-08-22

**Authors:** Hye Yoon Seol, Soojin Kang, Sungkean Kim, Jihoo Kim, Euijin Kim, Sung Hwa Hong, Il Joon Moon

**Affiliations:** 1 Department of Communication Disorders, Ewha Womans University, Seoul 03760, Republic of Korea; 2 Center for Digital Humanities and Computational Social Sciences, Korea Advanced Institute of Science and Technology, Daejeon 34141, Republic of Korea; 3 Department of Human–Computer Interaction, Hanyang University, Ansan 15588, Republic of Korea; 4 Department of Interdisciplinary Robot Engineering Systems, Hanyang University, Ansan 15588, Republic of Korea; 5 Department of Otolaryngology-Head and Neck Surgery, Soree Ear Clinic, Seoul 07560, Republic of Korea; 6 Hearing Research Laboratory, Samsung Medical Center, Seoul 16419, Republic of Korea; 7 Department of Otolaryngology-Head & Neck Surgery, Samsung Medical Center, Sungkyunkwan University School of Medicine, Seoul 03181, Republic of Korea

**Keywords:** hearing loss, evoked potentials, cochlear implants

## Abstract

**Background:** It has been reported in many previous studies that the lack of auditory input due to hearing loss (HL) can induce changes in the brain. However, most of these studies have focused on individuals with pre-lingual HL and have predominantly compared the characteristics of those with normal hearing (NH) to cochlear implant (CI) users in children. This study examined the visual and auditory evoked potential characteristics in NH listeners, individuals with bilateral HL, and CI users, including those with single-sided deafness. **Methods:** A total of sixteen participants (seven NH listeners, four individuals with bilateral sensorineural HL, and five CI users) completed speech testing in quiet and noise and evoked potential testing. For speech testing, the Korean version of the Hearing in Noise Test was used to assess individuals’ speech understanding ability in quiet and in noise (noise from the front, +90 degrees, and −90 degrees). For evoked potential testing, visual and auditory (1000 Hz, /ba/, and /da/) evoked potentials were measured. **Results:** The results showed that CI users understood speech better than those with HL in all conditions except for the noise from +90 and −90 degrees. In the CI group, a decrease in P1 amplitudes was noted across all channels after implantation. The NH group exhibited the highest amplitudes, followed by the HL group, with the CI group (post-CI) showing the lowest amplitudes. In terms of auditory evoked potentials, the smallest amplitude was observed in the pre-CI condition regardless of the type of stimulus. Conclusions: To the best of our knowledge, this is the first study that examined visual and auditory evoked potentials based on various hearing profiles. The characteristics of evoked potentials varied across participant groups, and further studies with CI users are necessary, as there are significant challenges in collecting and analyzing evoked potentials due to artifact issues on the CI side.

## 1. Introduction

Hearing loss (HL) refers to the impairment of auditory function, and numerous studies have reported its negative impacts on quality of life [1,2,3]. When HL occurs, individuals begin to experience difficulty perceiving and understanding speech, ultimately leading to communication breakdown. These breakdowns in communication can manifest in educational and occupational settings, affecting individuals’ performance. In addition, recent studies have reported a potential link between HL and dementia, leading to an increased focus on studying HL [4]. Therefore, early and appropriate interventions for HL are important. HL management or aural rehabilitation typically begins with hearing aids, which amplify sounds to improve audibility [5]. Healthcare professionals first program hearing aids based on individuals’ auditory characteristics [6]. Then, the individuals go through adjustment periods, during which they communicate in various situations, such as quiet and noisy places. Based on these experiences, the hearing aids are fine-tuned or adjusted during follow-up visits. If individuals do not receive much benefit from hearing aids, a cochlear implant (CI) could be considered. Similar to hearing aids, the primary goal of CIs is to enhance audibility by providing electrical stimulation, ultimately improving quality of life. CIs have been known to be effective for individuals for whom rehabilitation with conventional hearing aids was not effective [7,8,9]. Over time, more people have received benefit from CIs, as technological advancements in CIs have been made and CI candidacy has been expanded [10,11]. For example, there have been significant advancements in CI technology, transitioning from single-channel to multi-channel devices with varying numbers of electrodes. Additionally, features, such as beamforming and noise reduction, have been developed to enhance auditory perception in everyday environments. In 2008, a hybrid model combing a CI and a hearing aid was introduced. The design of CIs has also diversified, with behind-the-ear and off-the-ear models, reflecting ongoing improvements in both functionality and user convenience. However, it is important to note that CI use does not always lead to improvements in speech understanding [12,13,14,15,16,17,18,19], and individual variability in CI outcomes remains one of the most challenging issues in CI research. One of the well-researched benefits of CIs is in the area of speech recognition. Factors that can affect speech recognition include the duration of HL, the onset of HL, the position of the CI electrode array, the duration of auditory deprivation, and so on [16,18,20,21,22]. Generally, it is known that individuals who wear hearing devices early or have a shorter duration of HL tend to have better outcomes [23]. However, there are cases where individuals with similar demographic information and medical histories exhibit poor outcomes, and the underlying mechanisms for this are not well understood [12].

Currently, in clinical settings, speech performance can be examined using tests at the monosyllable, word, and sentence levels [24]. To reflect various communication environments, there are tools available that allow for testing not only in quiet conditions but also in noise conditions. In addition to these tests, electrophysiological testing, such as cortical auditory evoked potentials (CAEP), is also conducted to measure CI benefits at the central level. CAEPs refer to electrical activities from neurons in the auditory cortex [25]. They are typically recorded with electrodes placed on the scalp and have been greatly investigated, as they could objectively assess the functionality and maturity of the central auditory system [26]. For adults, the main components of the CAEPs are the P1–N1–P2 complex, which appear 50 to 200 ms after stimulation. P1 is the positive peak appearing after 50 ms, N1 is the negative peak appearing after 100 ms, and P2 is the positive peak appearing after 200 ms [27]. Research related to CAEP and HL has been conducted across various age groups, including children and the elderly, and across different hearing devices, such as hearing aids and CIs. Sandmann et al. (2012) recruited 22 individuals (11 with NH and 11 CI users with post-lingual HL) and explored their visual evoked potentials (VEPs), which are electrical signals evoked by visual stimulation. For analysis, the authors compared amplitudes and latencies of P100 (the same as P1, a positive peak occurring generally at 100 ms), N150, and P270. When comparing P100 VEP, CI users had a lower P100 VEP amplitude and shorter P100 latencies than those with NH, and recruitment of the right auditory cortex was observed in CI users [28]. For children with congenital HL, Sharma et al. (2002) reported that those with less than 3.5 years of HL showed P1 latencies within normal limits within 6 months of using a CI [29].

While there are studies that have explored electrophysiological characteristics in individuals with normal hearing (NH) and HL using CAEPs, most of these studies have focused on children, with relatively few examining adults. Additionally, there is a significant lack of research involving individuals using hearing aids or CIs, as well as those with diverse hearing characteristics. This study explores the P1 and N1 characteristics of individuals with various auditory characteristics. We hypothesized that individuals with HL would exhibit a larger amplitude than those with NH, and that CI users would show a smaller amplitude after CI surgery.

## 2. Materials and Methods

### 2.1. Participants

The inclusion criteria for this prospective cohort study, conducted from 2020 to 2023, included adults aged 19 years and older. The NH group included individuals with hearing test results showing thresholds of 25 dB HL or below at frequencies from 125 to 8000 Hz. The HL group included those with sensorineural HL above 30 dB HL based on the four-frequency pure-tone average (500, 1000, 2000, and 4000 Hz). The CI group comprised individuals with severe to profound HL scheduled for CI surgery. The exclusion criteria included individuals who had difficulty watching TV from a distance of 1 m and those with otological pathology and neurological and mental disorders. All experimental procedures were approved by Samsung Medical Center’s Institutional Review Board. Prior to testing, an informed consent document was obtained from the participants.

### 2.2. Pure-Tone Audiometry

Pure-tone audiometry was performed in a sound booth using insert earphones and an AudioStar Pro (Grason-Stadler, Eden Prairie, MN, USA) audiometer.

### 2.3. Speech Testing

The Korean version of the Hearing in Noise Test (K-HINT) is a speech-in-noise test widely used in South Korea. The K-HINT has a total of 240 sentences (20 sentences per list × 12 lists). The target sentences were presented through a loudspeaker located in front of the participants in a sound-treated booth using HINT pro 7.2 (Natus, Middleton, WI, USA). The participants were asked to listen to the sentences and then repeat them back to the tester. The testing was conducted in four conditions: quiet, noise from the front, noise from +90°, and noise from −90°. The presentation level was 65 dBA. In the conditions involving noise, the testing began at a 0 dB signal-to-noise ratio (SNR). If the participant correctly repeated the sentence, the level of the speech was decreased by 4 dB. If the participant incorrectly repeated the sentence, the speech level was increased by 2 dB. The K-HINT was performed twice, and the average was calculated for all participants.

### 2.4. CAEP Recording and Preprocessing

All recordings were conducted with the ActiveTwo BioSemi system (Amsterdam, The Netherlands). The electrodes were placed according to the 10–20 system. The electrodes were placed at Cz, Pz, Fz, T7, T8, O1, O2, and Oz. Reference electrodes were placed on the mastoids. Four additional electrodes were placed on the upper and lower part of the left eye and the outer canthi of both eyes for electro-oculograms. The sampling rate was 2048 Hz, and electrode impedances were kept below 5 kΩ. The acquired EEG data were filtered using a 1–30 Hz band-pass filter. Visual inspection was performed for movement artifacts. Then, the data were epoched from 100 ms pre-stimulus to 500 ms post-stimulus. For baseline correction, the epochs were deducted from the mean value of the pre-stimulus interval. Epochs including significant physiological artifacts (amplitude exceeding ±75 μV) at any electrodes were rejected. All EEG preprocessing steps and additional analysis procedures were carried out using MATLAB 2021 (Mathworks Inc., Natick, MA, USA).

### 2.5. Visual Evoked Potentials

For the VEP, the reversed displays of checkerboard patterns, which are widely used due to simplicity, were used. The stimuli consisted of black and white squares (10 × 10 pattern-reversal checkerboard) and were presented on a monitor using Neuroscan STIM2 (Charlotte, NC, USA). The stimulus interval was randomized between 900 ms and 1100 ms and the stimulus was repeatedly presented 500 times. The stimulus interval was 1000 ms. The participants were seated in a comfortable chair in a darkened room and asked to look at the center of the checkerboard image during the testing. The distance between the participant and the monitor was 1 m. After preprocessing the EEG data, peak detection of the elicited P100 component was performed. Trials were averaged at O1, Oz, and O2 electrodes, respectively. The most positive peak amplitude of the P100 component was defined between 60 and 200 ms, which was determined by the time interval between the zero crossings of the grand averaged waveform.

### 2.6. Auditory Evoked Potentials

AEPs in response to three stimuli (/da/, /ba/, and 1000 Hz) were recorded. Each stimulus had a duration of 170 ms and was repeatedly presented 300 times. The stimulus interval ranged from 900 ms to 1100 ms. The duration of the stimulus was 170 ms, and the stimulus interval was 1000 ms. The stimuli were presented though a speaker located 1 m from the participants with the presentation level of 65 dB, and white noise was presented in the opposite ear using an insert earphone at 45 dB. The participants sat on a comfortable chair and watched a movie without sound during the testing. After preprocessing the data for each stimulus type, the trials were averaged at Fz, Cz, Pz, T7, and T8 electrodes, respectively. The N100 component was elicited during the paradigms and the most negative peak amplitude of the N100 component was defined between the designated time windows. The time intervals between the zero crossings of the grand averaged N100 waveforms by each stimulus were used to determine the time windows. Specifically, the time window for the /ba/ stimulus was set between 90 and 190 ms, for the /da/ stimulus between 60 and 185 ms, and for the 1000 Hz tone stimulus between 60 and 185 ms after the stimulus onset.

## 3. Results

### 3.1. Participant Characteristics

A total of 16 participants were enrolled in the study. Characteristics of the CI users are described in Table 1. Among the participants, seven had NH, four had bilateral sensorineural HL, and five were CI users. The age range of the participants was from 23 to 67 years old, with a mean age of 44.1 years (SD = 14.2). The four-frequency pure-tone averages of the NH group were 7.1 dB in the right ear and 5.9 dB in the left ear. Individuals in the HL group had moderately severe sensorineural HL in both ears, and their pure-tone averages were 62.5 dB in the right ear and 59.7 dB in the left ear. In the CI group, the pure-tone averages were 59.0 dB in the right ear and 81.5 dB in the left ear. Among the CI group, two individuals (CI1 and CI2) had single-sided deafness in the right ear, and the four-frequency pure-tone averages were 15 and 11.2 dB for CI1 and CI2.

### 3.2. Speech Performance

The average K-HINT scores in quiet were 9.2, 61.4, and 31.7 dBA for the NH, HL, and CI groups. When the noise was presented from the front, the average scores were −4.4, 3.4, and 0.3 dB SNR for the NH, HL, and CI groups. In the noise from +90° condition, the average scores were −14.5, −0.3, and 2.6 dB SNR for the NH, HL, and CI groups. Lastly, in the noise from −90° condition, the average scores were −12.4, −1.4, and −0.2 dB SNR for the NH, HL, and CI groups. In all test conditions, the NH groups showed the best performance. Comparing the HL and CI groups, the CI users performed better than those with HL in all conditions except for the noise from +90° and −90° conditions.

### 3.3. Visual Evoked Potentials

**Figure 1:**
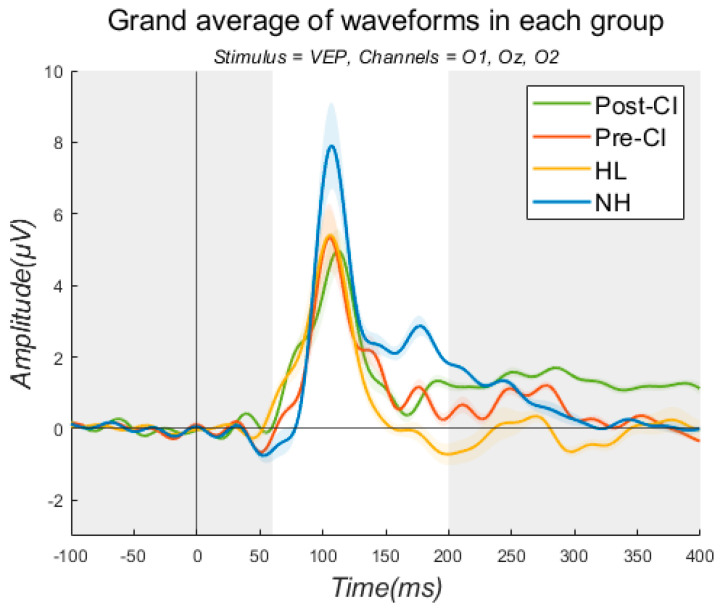
Grand average waveforms for VEP.

Figure 1 illustrates the grand average waveforms for VEP. Only the P1 amplitudes were assessed. Table 2 describes the average P1 amplitudes for all three groups. For O1, the P1 amplitudes for the NH and HL groups were 5.7 and 5.3 µV. For the CI group, the P1 amplitude before implantation was 5.6 µV, and after implantation it was 4.3 µV. For Oz, the amplitudes were 7.6 and 6.1 µV for the NH and HL groups. The amplitudes were 8.2 and 5.5 µV in the pre- and post-CI conditions for the CI users. Lastly, for O2, the P1 amplitudes were observed to be 7.4 and 5.7 µV for the NH and HL groups. The P1 amplitudes were 7.8 and 5.3 µV before and after implantation for the CI users. For the CI group, overall reductions in the P1 amplitudes were observed for all channels after implantation. The NH group showed the largest amplitudes, followed by the HL group and the CI group (post-CI).

### 3.4. Auditory Evoked Potentials

**Figure 2:**
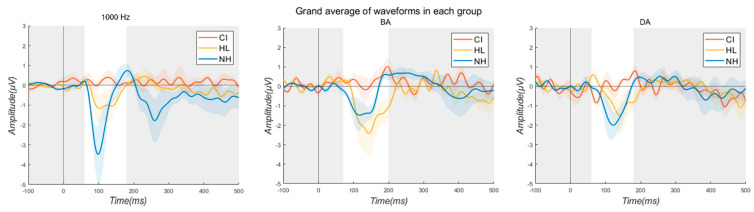
Grand average waveforms for AEP.

Table 3 describes the average N1 peak amplitudes for all three groups in each stimulus condition. Figure 2 illustrates the grand average waveforms for AEP. When comparing the average N1 peak amplitude between the NH and HL groups, the N1 peak amplitudes at 1000 Hz were found to be smaller in the HL group. For /ba/, the N1 peak amplitude in the HL group was larger at all electrodes except T8. For /da/, the N1 peak amplitude was larger in the NH group at the Fz, T7, and T8 electrodes. The smallest N1 peak amplitude was observed in the pre-CI condition for all stimuli except for /da/ at the T8 electrode.

## 4. Discussion

This study investigated speech understanding ability as well as P1 and N1 characteristics in individuals with NH and HL, and CI users. The results revealed that, in terms of speech recognition, the NH listeners were able to understand speech better than the HL and CI users. Comparing between the HL and CI groups, the CI group’s speech performance was better than the HL group except for in two conditions (+90° and −90°). Regarding the P1 and N1 responses, P1 (VEP) and N1 (AEP) amplitudes were compared for all groups. Compared to the NH group, the HL and CI groups showed smaller P1 amplitudes in response to VEP. A comparison of the pre- and post-CI conditions showed that implantation led to smaller P1 amplitudes. For AEP, the smallest N1 amplitude was generally observed in the pre-CI condition. When comparing the average N1 peak amplitude between the NH and HL groups, the HL group showed smaller N1 peak amplitudes at 1000 Hz. For the /ba/ stimulus, the N1 peak amplitude was greater in the HL group at all electrodes except T8. Conversely, for the /da/ stimulus, the NH group had larger N1 peak amplitudes at the Fz, T7, and T8 electrodes. Findings of the study are in line with previous studies, to some extent, showing that HL led to poor speech performance and smaller P1 and N1 amplitudes [28,30,31,32,33,34]. Campbell and Sharma (2014) examined the VEP response in nine individuals with NH and eight individuals with mild to moderate HL, and found that the P1, N1, and P2 amplitudes were larger in the HL group [33]. Harkrider et al. (2006) investigated N1–P2 cortical evoked responses in 11 young adults and 10 older adults with NH, and 10 older adults with mild-to-moderate HL. They found that young adults with NH showed the smallest N1 amplitudes, while among the older adults with NH and HL, the older adults with HL exhibited larger N1 amplitudes [34]. This study is meaningful, as it investigated the P1 and N1 characteristics in individuals with various hearing characteristics, including those with NH, HL, CI users, and specifically those with single-sided deafness using a CI. However, many aspects need to be improved to explain the electrophysiological characteristics in such diverse groups. Firstly, similar to other studies, the small sample size in this study made it difficult to generalize the findings, so further research with a larger sample size is necessary. Regarding various hearing profiles, it would be meaningful to explore the changes in electrophysiological characteristics based on not only single-sided deafness but also various etiologies, durations of HL, types of hearing devices, and so on. While this study used only eight electrodes, employing more electrodes could allow for a more detailed examination of brain-region-specific characteristics. It is also important to investigate brain characteristics at different time points in terms of brain plasticity. Stropahl et al. (2017) noted that cortical change patterns may vary depending on the degree of HL, and how these patterns change following sensory restoration via CI is not well understood [18]. The authors emphasized the need for prospective longitudinal studies with various time points to understand the factors driving cortical changes and the nature of these patterns. Therefore, including pre-CI as well as post-CI conditions at intervals such as three, six, and nine months, and so on, would be beneficial. Lastly, while electrophysiological components include not only P1 and N1 but also P2, P300, and others, this study was limited by artifacts, allowing comparison only of the P1 amplitude in VEP and the N1 amplitude in AEP. Artifacts in EEG conducted on CI users have been a longstanding issue, and studies are ongoing to address this [35,36]. Intartaglia et al. (2022) mentioned the importance of the development of reliable techniques of EEG artifact removal since the artifacts caused by CIs could distort EEG responses. However, even though various methods have been employed in past research, it is still difficult to determine the best EEG artifact removal technique, as there is a lack of documentation and agreement.

In summary, research studies investigating electrophysiological characteristics in individuals with various types and degrees of HL has shown mixed findings. This variability can be attributed to factors, such as the limited amount of research in this area, small sample sizes, and methodological differences, including stimuli presentation levels and the signal-to-noise ratio (SNR) [12,37,38,39]. Regarding the stimuli presentation level, in this study, the presentation level of all stimuli was fixed at 65 dBA. Several studies have mentioned that different CAEP response characteristics can be observed depending on the stimulus intensity and SNRs [38,39]. Gurkan et al. (2023) examined CAEP responses in three groups (NH, mild HL, and moderate HL) with a stimulus (/g/) presented at 10, 20, and 30 dB SNRs [38]. The authors reported that those with moderate HL showed decreasing N1–P2 responses as SNRs decreased. Considering that everyday communication environments involve various SNRs and that stimulus levels are perceived differently depending on the type and degree of HL, it is essential to take hearing status into account when determining the presentation level of the stimulus. Another methodological difference is the criteria used to distinguish participant characteristics. While this study did not divide CI users into well-performing and poor-performing groups based on speech performance, some studies have categorized participants in this way, leading to differing findings [31,40]. Kim et al. (2016) investigated the VEP characteristics of 14 CI users and 12 NH listeners. When dividing the CI group into poor performing and well performing, it was found that the poor-performing CI group showed larger P1 amplitudes in the right temporal cortex and smaller P1 amplitudes at electrodes near the occipital cortex [31]. Doucet et al. (2006) also investigated VEP characteristics in 13 CI users and 16 NH listeners. Similar to Kim et al. (2016), this study divided the CI users into poor-performing and well-performing groups. While no differences in P1 and N1 amplitudes were observed, the study found that the P2 amplitude was significantly larger at the occipital site [40].

As for future research, Pisoni et al. (2018) mentioned that future studies related to CI should focus more on individuals with poor outcomes rather than those with good outcomes [12]. They noted that, aside from device checks and commonly conducted audiological testing in clinical settings, there is a lack of evaluation and intervention protocols for individuals with poor outcomes. They also suggested that additional assessments for cognitive domains should be incorporated. Since each individual with HL has unique characteristics, it is essential to go beyond the conventional audiological testing and include assessments of cognitive and psychosocial domains to accurately understand their characteristics at peripheral and central levels.

## Figures and Tables

**Table 1:** Participant characteristics.

Group	Sex	Age	Four-Frequency Pure-Tone Average (Right/Left in dB)	Etiology of HL	Duration of HL (mos)	CI Side	Device
NH1	F	32	7.1/5.9	N/A	N/A	N/A	N/A
NH2	F	23					
NH3	F	30					
NH4	F	23					
NH5	M	44					
NH6	F	43					
NH7	M	26					
HL1	M	54	62.5/59.7	Sudden	192	N/A	N/A
HL2	M	62		Unknown	24		
HL3	F	63		Unknown	144		
HL4	F	61		Sudden	288		
CI1	M	42	59.0/81.5	Chronic otitis media	48	L	KANSO 2
CI2	F	51		Sudden	24	L	RONDO 2
CI3	F	56		Sudden	96	R	KANSO 2
CI4	F	36		Unknown	240	L	KANSO 2
CI5	F	48		Unknown	240	L	KANSO 2

N/A: Not available.

**Table 2:** Average P1 amplitudes for Oz, O1, and O2 for the groups.

Group	Average Amplitudes (µV)		
	Oz	O1	O2
NH	5.7	7.6	7.4
HL	5.3	6.1	5.7
Pre-CI	5.6	8.2	7.8
Post-CI	4.3	5.5	5.3

**Table 3:** Average N1 amplitudes for 1000 Hz, /ba/, and /da/ for the groups.

Stimulus	Group	Average Amplitudes (µV)				
		Fz	T7	Cz	T8	Pz
1000 Hz	NH	−5.9	−2.6	−5.1	−3.2	−3.1
	HL	−2.5	−1.4	−2.3	−0.7	−1.6
	Pre-CI	−0.2	−0.9	0.1	−0.3	0.1
/ba/	NH	−3.3	−1.9	−3.0	−2.1	−2.0
	HL	−4.3	−2.3	−4.3	−1.5	−2.8
	Pre-CI	−1.0	−0.7	−0.9	−0.4	−0.6
/da/	NH	−3.3	−1.6	−3.3	−2.3	−2.2
	HL	−3.2	−1.4	−3.4	−1.3	−2.3
	Pre-CI	−1.1	−1.0	−1.1	−1.7	−1.2

## Data Availability

The data that support the findings of this study are available from the corresponding author upon reasonable request.
